# Expanding an expanded genome: long-read sequencing of *Trypanosoma cruzi*

**DOI:** 10.1099/mgen.0.000177

**Published:** 2018-04-30

**Authors:** Luisa Berná, Matias Rodriguez, María Laura Chiribao, Adriana Parodi-Talice, Sebastián Pita, Gastón Rijo, Fernando Alvarez-Valin, Carlos Robello

**Affiliations:** ^1^​ Laboratory of Host Pathogen Interactions-UBM, Institut Pasteur de Montevideo, Montevideo, Uruguay; ^2^​ Sección Biomatemática - Unidad de Genómica Evolutiva, Facultad de Ciencias-UDELAR, Montevideo, Uruguay; ^3^​ Departamento de Bioquímica, Facultad de Medicina-UDELAR, Montevideo, Uruguay; ^4^​ Sección Genética, Facultad de Ciencias-UDELAR, Montevideo, Uruguay

**Keywords:** *Trypanosoma cruzi*, PacBio, whole genome sequencing, Chagas disease

## Abstract

Although the genome of *Trypanosoma cruzi*, the causative agent of Chagas disease, was first made available in 2005, with additional strains reported later, the intrinsic genome complexity of this parasite (the abundance of repetitive sequences and genes organized in tandem) has traditionally hindered high-quality genome assembly and annotation. This also limits diverse types of analyses that require high degrees of precision. Long reads generated by third-generation sequencing technologies are particularly suitable to address the challenges associated with *T. cruzi*’*s* genome since they permit direct determination of the full sequence of large clusters of repetitive sequences without collapsing them. This, in turn, not only allows accurate estimation of gene copy numbers but also circumvents assembly fragmentation. Here, we present the analysis of the genome sequences of two *T. cruzi* clones: the hybrid TCC (TcVI) and the non-hybrid Dm28c (TcI), determined by PacBio Single Molecular Real-Time (SMRT) technology. The improved assemblies herein obtained permitted us to accurately estimate gene copy numbers, abundance and distribution of repetitive sequences (including satellites and retroelements). We found that the genome of *T. cruzi* is composed of a ‘core compartment’ and a ‘disruptive compartment’ which exhibit opposite GC content and gene composition. Novel tandem and dispersed repetitive sequences were identified, including some located inside coding sequences. Additionally, homologous chromosomes were separately assembled, allowing us to retrieve haplotypes as separate contigs instead of a unique mosaic sequence. Finally, manual annotation of surface multigene families, mucins and trans-sialidases allows now a better overview of these complex groups of genes.

## Data Summary

The genome assemblies and annotation of TCC and Dm28c have been deposited in GenBank with the accession numbers: PRJNA432753 and PRJNA433042, respectively. Raw sequences of TCC and Dm28c have been deposited in GenBank with the accession numbers SRP134374 and SRP134013, respectively. Supporting Data tables are available on Figshare data: https://figshare.com/s/38da1cde5667299b3a1e. We confirm that all supporting data, code and protocols have been provided within the article or through supplementary data files.

**Impact Statement**

We present the assembled and annotated genomes of two *Trypanosoma cruzi* clones, the hybrid TCC and the non-hybrid Dm28c, obtained using PacBio technology. The complications associated with this genome (assembly fragmentation and collapse of repetitive sequences) were basically solved. These improved assemblies permitted us not only to accurately estimate copy numbers of tandemly arrayed genes and multigene families but also allowed the unambiguous identification of many single-copy genes. Reliable information on the latter is required to conduct functional studies based on gene knock-out, a fundamental source of information to determine which genes are essential and to find new drug targets for Chagas disease.

We found that the genome of *T. cruzi* is compartmentalized in a isochore-like manner, containing a ‘core compartment’ and a ‘disruptive compartment’, which exhibit significant differences in GC content and gene composition, the former being GC-poorer and composed of conserved genes and the latter enriched in trans-sialidases (TS), mucins and mucin-associated surface protein (MASP) genes.

Additionally, many homologous chromosomes were separately assembled (haplotypes), and some homologous recombination events could be identified. The availability of high-quality genomes opens new possibilities to conduct analysis on this parasite that require high degrees of precision of genomic data (e.g. allelic exclusion, population genomics).

## Introduction

The year 2005 represents a landmark in the study of trypanosomatid biology with the simultaneous publication of the *Leishmania major, Trypanosoma brucei* and *Trypanosoma cruzi* genomes; three species considered to be representative of the parasitic diversity of the group [1–3]. The new information obtained from these genomes opened a new era that allowed researchers to conduct studies with unprecedented comprehensiveness. Several new types of analyses, such as large-scale comparative genomics, aimed at understanding the common evolutionary basis of parasitism and pathogenesis, or searching for new vaccine candidates and drug targets [4, 5] were made possible. These three genomes, however, were published with very different degrees of finishing. The genome of *L. major* was precisely assembled and annotated. The *T. brucei* genome was also of very good quality but was mostly focused on megabase chromosomes, whereas minichromosomes were not included. Conversely, the assembly of the *T. cruzi* genome (CL Brener strain) was extremely fragmented (4098 contigs) with very few contigs exceeding 100 kb in size. In fact, only 12 contigs met this threshold and none was larger than 150 kb [3]. Despite this degree of fragmentation, this draft genome was highly valuable because it led to the identification of several novel species-specific multigene families and gave, for the first time, a draft overview of the genome architecture of *T. cruzi*. This draft, also made it possible to identify the vast majority of conserved trypanosomatid genes, as indicated by the fact that very few genes conserved between *Leishmania* and *T. brucei* were not found in the draft *T. cruz*i genome [4]. Most likely this can be attributed to the fact that genes are short in trypanosomatids (since they lack introns), hence even moderately small contigs can contain complete coding sequences (CDS). The genome sequences of additional *T. cruzi* strains Dm28c [6], Sylvio X10/1 [7], and *T. c. marinkellei* [8] have been reported since this initial publication using new sequencing technologies, such as Roche 454 alone or in combinations with other methodologies (Illumina and Sanger). A common feature of all the assemblies reported for these strains is again high fragmentation, which was even higher than that originally reported for CL Brener.

To tackle the problem of assembly fragmentation, Weatherly *et al.* [9] used complementary sources of data to scaffold *T. cruzi* (CL Brener strain) contigs, aiming to recover full-length chromosome sequences. Their approach used a combined strategy based on sequencing bacterial artificial chromosome (BAC) ends, as well as their co-location on chromosomes and synteny conservation with *T. brucei* and *Leishmania* chromosomes. This strategy enabled these authors not only to obtain an assembly that represented a substantial improvement in comparison to previous versions of the genome but also to reconstruct chromosomes (with a few gaps), with only a relatively minor portion of the genome remaining unassigned to chromosomes. Nonetheless, the issue of assembly fragmentation is a limitation that poses a number of complications for diverse types of analyses that require high precision.

Third-generation sequencing technologies are particularly suitable for addressing the challenges associated with the peculiarities of the *T. cruzi*'s genome since they allow sequencing reads of more than 15 kb in average length, and many much longer can be obtained. This opens up the possibility of directly determining the full sequence of large clusters of repetitive sequences (without collapsing them), as well as determining the single-copy sequences that flank both sides of these clusters. The collapse of tandemly arrayed gene copies erases any variability that these copies might eventually exhibit, thus precluding any assessment in which this variability may be relevant (for instance intra-cluster differential gene expression). As a consequence, assembly fragmentation is largely overcome. In fact, using this technology has made it possible to obtain much better quality genome assemblies (compared with pre-existing ones) in other parasitic protozoa that also have highly repetitive genomes, such as *Plasmodium coatneyi* [10]. More recently the full length chromosome sequences (without gaps and collapsed segments) were obtained for *Plasmodium malariae* [11]. Furthermore, this technology allows obtaining separated assemblies of homologous chromosomes, such that haplotypes can be retrieved as separated contigs/scaffolds instead of a unique mosaic sequence. This feature is particularly desirable in genomes with a high level of heterozygosity, as in the case of some *T. cruzi* hybrid strains that have arisen from relatively divergent ancestors, in which the divergence between haplotypes is higher than 5 % [3]. Needless to say, a single mosaic assembly, representing a mixture of two parental sequences, can produce distortions in several types of analyses, such as detection of recombination or aneuploidies.

In order to contribute to the genomic information on this parasite, we report here the genome sequence of two *T. cruzi* strains, TCC and Dm28c, obtained using long-read sequencing. Our results significantly improve the quality of the genome assembly and annotation available for this parasite, implying more precise estimations of genome sizes, gene copy numbers and repetitive sequence distribution. These results also reveal multiple unique aspects of genome architecture previously overlooked, such as whole-genome compartmentalization into regions of different base composition and biased distribution of genes.

## Methods

### Parasites and DNA isolation

*T. cruzi* strains Dm28c [12] and TCC [13], were used throughout this work. Epimastigotes were grown in liver infusion tryptose (LIT) medium supplemented with 10 % fetal calf serum at 28° C; total DNA was extracted using the Quick DNA Universal kit (Zymo Research).

### Sequencing

PacBio library preparation and sequencing were done by the University of Washington PacBio Sequencing Services (Washington, USA). Briefly, DNA was mechanically fragmented using a Covaris g-TUBE device, and concentrated with AMPure PB magnetic beads. The final long-insert PacBio libraries were size-selected for fragments larger than 10 kb using the BluePippin device.

A total of eight Single Molecular Real-Time (SMRT) cells were used, five for TCC and three for Dm28c, yielding 751 460 and 601 168 raw reads, respectively. Subreads were obtained using the SMRT Analysis RS.Subreads.1 pipeline (minimum polymerase read quality=0.85; minimum polymerase read length and minimum subread length=500 bp). An Illumina MiSeq platform was used with a paired-end library (2×150 cycles). Briefly, a Nextera XT (Illumina) library preparation kit was used with 1 ng total DNA according to the manufacturer's instructions. Index primers were added to each library to allow sequence multiplexing. After 12 PCR cycles, the final library was purified with AMPure XP (Benchman) and quantified with the Qubit dsDNA HS assay kit (Invitrogen). Quality and length of the library were assessed with the Agilent high-sensitivity DNA kit (Agilent) using the 2100 Bioanalyzer (Agilent). The raw reads obtained from the two genomes were deposited at Sequence Read Archive (SRA) NCBI repositories (Accession IDs: SRP134374 and SRP134013).

### Genome assembly

The assembly was performed using the SMRT Analysis tools implemented in the Hierarchical Genome Assembly Process (HGAP) pipeline V3 [14]. It was run with the default parameters modifying only the expected genome size, which was set to 110 Mb, generating 1978 and 1143 contigs with a shortest sequence length at 50 % of the genome (N50) of 73 kb and 129 kb for TCC and Dm28c, respectively. Subsequently the scaffolding pipeline IPA was applied [15], specifically those scripts aimed to merge overlapping contigs using the assembled output and Illumina paired-end reads. This pipeline starts by self-mapping the contigs using megablast, with the parameter of word-size set at 40 and an e-value of 1e−80. Self-contained contigs and those with an identity below 99 % and shorter than 500 bp were discarded for further analysis. Then Illumina reads were mapped over the remaining sequences using the alignment software SMALT. Finally, if there are enough pairs of Illumina reads where each member of the pair maps to two different contigs, it can be argued that the pair comes from the same contiguous sequence. This information was used to merge contigs by their ends. The final assemblies were of 86.7 and 53.2 Mb with a N50 of 265 and 318 kb for TCC and Dm28c respectively. Both genomes and their annotations were deposited at the NCBI repository (BioProjects PRJNA432753 and PRJNA433042).

### Genome annotation

For the annotation of the coding sequences we extracted from each assembled genome the open reading frames of at least 150 amino acids in length, between a start and a stop codon, using the getorf tool from the EMBOSS suite [16]. These sequences were mapped using blastp [17] against a database of kinetoplastid proteins from TriTrypDB using as cut-off an e-value of 1e−10. The database of kinetoplastid proteins consists of a curated collection of CDS from various species of the genera *Trypanosoma* (excluding *Trypanosoma grayi* and *Trypanosoma rangeli*)*, Leishmania, Leptomonas, Crithidia* and *Endotrypanum.*

For practical purposes the annotation was divided into several steps. First the surface multigene families i.e. mucin-associated surface proteins (MASPs), mucins, retrotransposon hot spot proteins (RHS), zinc metalloprotease gp63 (GP63), trans-sialidases (TS), dispersed gene family protein 1 (DGF-1) and the recently identified Trypomastigote, alanine, serine, valine proteins (TASV) [18] were annotated using a stricter cut-off, an e-value of 1e−30. Then we annotated the genes conserved among kinetoplastids. In this step we chose the best hit in *T. cruzi* CL-Brener Esmeraldo and non-Esmeraldo strains, and the best hit outside *T. cruzi*. If there were hits only outside *T. cruzi* these ORFs were also annotated with the best two hits. The selection of the best high-scoring pairs (HSP) was performed using a personalized script that scanned for the lowest possible e-value with a meaningful description. If the only description available was ‘hypothetical protein’ or a similar non-descriptive tag, then the lowest e-value was selected. For annotating short proteins, those smaller than 150 amino acids, we extracted the open reading frames of between 50 and 150 amino acids generating a massive amount of sequences that were subsequently mapped only against a *T. cruzi* database by blastp searches (e-value 1e−03, identity >80 % and query coverage >80 %). Initital methionines were verified using RNA-seq data. Specifically, *T. cruzi* RNA-seq reads were downloaded from the SRA repository, bioproject PRJNA251583 [19], and the reads containing a spliced leader sequence were identified. The spliced leader segment was trimmed from these reads and the remaining segment was mapped to the *T. cruzi* genome to identify splice-acceptor sites (further details in [20]). Once the splice-acceptor sites were determined, we annotated as the initiation codon, the in-frame ATG triplet closest to (and downstream of) the splice-acceptor site. Additional information was included in the annotation by predicting signal peptide (SP), using SignalP v4.1 [21], glycosylphosphatidylinositol (GPI) anchor, predicted by predGPI [22], and trans-membrane domains, using TMHMM v2.0 [23].

A curated non-coding RNAs and interspersed repeats database (from TriTrypDB and NCBI) was used for the annotation of these elements. The sequences in these databases were mapped using blastn against the assembled contigs, then filtering the results and keeping only those hits which mapped more than an 80 % of the length of the queried sequence with at least an 80 % identity. An overview of the workflow is presented in Fig. S1 (available in the online version of this article).

Customized scripts were written in Python [24], Perl [25] and R [26]. Alignments of short reads (Illumina and 454 reads) were performed with Bowtie2 [27] and Rsubreads (from within R [28]). Samtools utilities [29] were used to manipulate alignments and perform coverage analyses

### Contigs comparison

#### Dotplot view

A self-comparative mapping of each contig, showing the results in a dotplot, was performed with blastn. We used the YASS web server [30] with the default options to create these plots.

#### Circos view

To assess the similarities between contigs of each assembly, we run a blastn of all-against-all the contigs longer than 50 kb. For practical purposes we generated two tables, one with HSPs longer than 10 kb and other with HSPs between 5 and 10 kb. To graphically represent these data and avoid an entangled plot with an over-representation of hits from multigene families, the hits from these sequences were removed from the final results. The mappings between contigs are shown in a circular layout using the Circos software [31]. In these plots (Fig. 2d and http://bioinformatica.fcien.edu.uy) the target sequence is shown at 12 o'clock and extends clockwise proportional to its length. The ten contigs with the largest overall mappings were selected, and are represented according to their length in the plot. The genomic similarities of the target sequence and the contigs are represented as wide lines that show the length of the mappings and their coordinates and share the same color with the contig of origin.

### The web interface

An online genome browser was developed (http://bioinformatica.fcien.edu.uy/cruzi) where the whole annotation is displayed for contigs longer than 50 kb. Each contig is shown as a long rectangular block where genes and sequences of interest are represented as rectangles of different colors according to their classification. The width and position of each rectangle is proportional to their length and coordinates in the contig. Each represented contig has at the bottom a smaller rectangle that indicates the direction 5′−3′: sense (red) or antisense (green). In the visualization interface the annotation is divided into different categories. The CDSs are divided according to the main multigene family they belong to, conserved genes are divided between those with a meaningful description and those annotated as hypothetical proteins. The repeated elements are also classified as tandem, interspersed repeats or satellite. Each category is indicated by a different color and can be displayed or hidden as a whole using the check boxes in the upper part of the page. When the cursor is over an annotated sequence it displays a tooltip with a description of the best blast hits and hyperlinks to the nucleic and amino acidic sequence if possible. Under each contig there is a white rectangular block with more information. There are hyperlinks to the contig sequence in fasta format. At the bottom-right of the page there are five buttons, two with the magnifying glass allow zooming in and out in the sequences, the one with the stripes allows changing of views between the standard view and a six frames view, and the text box and binoculars allows searching for sequence descriptions within the page. It is also possible to extract a customized nucleotide sequence from the contig by clicking at the start and end of the desired area. After doing this an emergent window appears at the bottom of the page, allowing correction of the coordinates and retrieval of the genomic sequence.

### Chromosome and contig visualization

Besides the developed web interface, we used ACT [32] and ARTEMIS [33] to visualize and compare contigs from our assembly and previous assemblies. Both software packages use the output of the blastn search and the contigs in fasta format. blastn searches for these comparisons were performed with a stringent e-value <1e−200. IGV [34] software was used to visualize the alignments of reads over the assemblies (including Illumina, 454 or PacBio reads of different sources).

### Manual curation of surface multigene families annotation

The annotation of surface multigene families was based on the coding sequences identified by the annotation pipeline. Furthermore, we manually inspected the sequences, analyzing specific characteristics relative to their function.

For mucins we use the length, the number of repetitions of threonine tandems, the presence of conserved sequences within the SP, the region surrounding the GPI and the S, T and P amino acid composition [35]. The identification of conserved N- and C-terminal domains as well as characteristic sequences and tandem repeats present in mucins were performed with custom scripts. Genes without a clear signal peptide and GPI anchor signal, but containing other mucin properties were designated as pseudogenes (most of them corresponded to genes that had lost the N- or C- terminal extremes). Finally, analysis of RNA seq data [19, 36] and correction of the initial methionine were performed where applicable.

For trans-sialidase sequences we considered the length, the presence of VTV and ASP box motifs, the GPI anchor signal probability and the presence of a SP sequence (using the same software as above). SXDXGXXTW and VTVxNVxLYNR sequences [37] were searched using PatMatch [38] and the output parsed with customized scripts.

For MASP genes, we consider the presence of highly conserved domains. N-terminal domain MAMMMTGRVLLVCALCVLWCG and C-terminal domain GDSDGSTA VSHTTSPLLLLLVVACAAAAAVVAA [3, 39] were searched using HHsearch [40]. Genes having only one domain were annotated as pseudogenes.

Fragmented DGF-1 pseudogenes were reconstructed into a single sequence. The partial pseudogenic ORFs were merged and the “full length” pseudogene thus obtained was compared with a functional copy used as template.

### Gene cluster analyses

Protein-coding genes were clustered into gene families using Markov Cluster Algorithm (MCL) [41] with blastp −log e-values (e−20). A fairly stringent inflation value which determines the granularity (or size of the output clusters) of 4 was used. A customized script was used to parse the clusterization output and generate the final results. Clusterization was performed to identify gene families, novel gene families and single copy genes.

### Characterization of transposable elements

Canonical complete elements for each transposable element (TE) were used to perform queries against the genomes using blastn (e-value 1e−10, identity >80 %, query coverage >90 %). Non autonomous elements (SIRE and NARTc) were filtered when mapped into the parental elements (VIPER and L1Tc). L1Tc was also identified in the CL Brener assembly (from TriTrypDB v28 *T. cruzi* CL Brener*, T. cruzi* CL Brener Esmeraldo-like and *T. cruzi* CL Brener non-Esmeraldo-like) and the Sylvio X10/1 assembly (from NCBI BioProject PRJNA40815) with same parameters. A total of seven L1Tc elements were identified in CL Brener and 156 in Sylvio X10/1. Multiple alignments were performed with MAFFT [42] using the e-ins-i Iterative refinement method. Phylogenetic trees were computed by PhyML 3.1 [43] through the SeView platform [44]. A maximum likelihood tree based on the GTR+i model was reconstructed with 1000 bootstrap replications. Final representation was performed with iTOL [45].

### Characterization of tandem repeats

To identify and annotate tandem repeats we used the Tandem Repeats Finder software [46] with the parameters of minimum alignment score of 20, maximum period size of 2000 and an alignment score of 2 for a matching base and −7 for mismatches and insertions and deletions (indels). All entries with at least ten repeated periods of at least three nucleotides in length were considered for analysis. The results that share an overlap over 80 % in both sequences were merged and reported as one. The grouping of these sequences was done in several steps. First, we used personalized scripts to generate all the variants of a period and scan the results to group the identical ones. This strategy is better suited to grouping short periods without internal variability.

The second step of clusterization consisted of creating a multifasta file with a sequence from each period and grouping them using blastclust with the parameters -S80 -L0.8 -bT that restricts the results to those that share at least 80 % of the sequence length with and 80 % of identity for both sequences. This grouping step is mainly aimed at clustering longer sequences with few indels or mismatches.

For further grouping, a third step was used, where each sequence was extended by repeating the period up to at least 300 bp. Then a self-comparative blast was performed and results with an identity lower than 80 % were discarded. Using a personalized script, for each sequence all the HSP were summed and if this sum was at least an 80 % of the target sequence, they were grouped. This step is particularly useful for grouping sequences where the shortest reported period is in fact a multiple of a shorter one of another cluster. The final output is a table with the coordinates of each tandem repeat and the group they belong. An overview of the workflow is presented in Fig. S2

## Results and Discussion

### Genome assembly

To obtain a more complete assembly of the complex *T. cruzi* genome using the PacBio technology, library preparation included fragmentation and size selection with a cut-off of 10 kb. This size threshold also prevents the inclusion of minicircles, whose presence leads to a substantial reduction in sequencing depth of nuclear genomic DNA (they represent about 20 % of total DNA). To include both clinically and evolutionarily relevant strains, we chose to sequence the hybrid TCC strain (TcVI), derived from Tulahuen 2 and closely related to CL Brener [13], and the non-hybrid strain Dm28c (TcI) [12]. It should be mentioned that TcI is the most abundant DTU and has a wide distribution in the Americas, evolving separately from a common ancestor [47] while TcVI, present in several countries of South America, is a hybrid of the two distinct lineages TcII and TcIII [48].

**Fig. 1.**
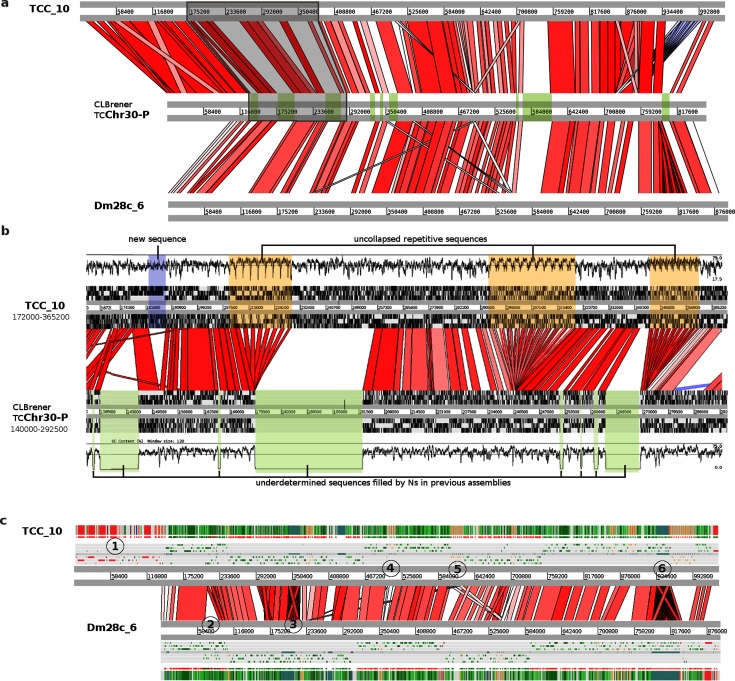
Chromosomal assembly improvements. (a) ACT alignment of homologous chromosomes from three strains: TCC (contig TCC_10), Dm28c (contig Dm28c_6) and CL Brener (chromosome TcChr30-P). Previously undetermined sequences filled by Ns in CL Brener are marked in green. (b) Magnification of a fragment of a (boxed and shadowed in grey). The six frames and the DNA G+C content of each chromosome are plotted. Previously collapsed repetitive sequences (boxed in orange) are disaggregated in the new assembly. **c)** Visualization of the alignment of the same homologous chromosome showing additional details in TCC and Dm28c. The color patterns in the annotation bars (bottom and top-most horizontal stripped bars) correspond to the annotation as they appear in the web interface (DGF1 in red, GP63 in orange, RHS in brown, conserved genes in green). The six reading frames are also shown. (1) Terminal DGF-1 gene cluster present only in TCC. (2) Non-homologous region present only in Dm28c. (3) Repetitive region present in both strains. (4) Expansion of a GP63 cluster in TCC (four copies versus two copies in Dm28c). (5) Strain-specific amplifications of two different genes. There are seven GP63 copies (orange strips on the top annotation bar) in TCC but only one in Dm28c; moreover Dm28c contains four RHS copies in the same region. (6) Repetitive element present in both genomes having fewer copies in TCC (20 copies in TCC and 44 copies in Dm28c). The segment is followed by another strain-specific amplification consisting of a cluster of 14 GP63 genes in TCC and only one copy in Dm28c.

After filtering by read quality, we obtained 5.2 and 4.0 Gb of sequences comprising 343 384 and 261 392 reads for TCC and Dm28c, respectively (Table 1). The average read length was 16 kb for both strains, and the longest reads were larger than 60 kb (Fig. S3). The availability of such long reads is essential to disentangle the sequence repetitions; a hallmark of *T. cruzi*. HGAPv.3 assembler [49] was used to correct and assemble the initial reads. IPA [15] was used to collapse overlapping contigs and cleanup the assembly incorporating Illumina reads. The length of the reads enabled us to directly obtain the full sequences of large clusters of repetitive sequences without collapsing them, as well as to determine the non-repetitive sequences flanking those repeats. Illustrative examples of how PacBio reads allowed us to overcome some of the common complications associated with *T. cruzi* assemblies, fragmentation and collapse of repeats, are presented in Fig. 1. The figure shows the comparison between chromosome 30 P from CL Brener (assembly reported in [9]) with TCC and Dm28c contigs reported here using long reads. In order to build the virtual chromosome 30 P (from CL Brenner) Weatherly *et al*. [9]connected many contigs by segments of unknown sequence (filled with N letters, shadowed in green in Fig. 1a). These ‘N regions’ were fully resolved in our assemblies, both in TCC and Dm28c (Fig. 1a). It is worth noting that the lengths of ‘N regions’ were precisely estimated by Weatherly *et al*. (Fig. 1).

**Table 1.** Summary of the assembly and annotation of *T. cruzi* TCC and *T. cruzi* Dm28c genomes

**Assembly properties**	**TCC**	**Dm28c**
Total reads	751 460	601 161
Filtered reads	343 384	261 392
Read N50	20 939	21 011
Total base pairs	5 200 759 160	4 037 444 749
Coverage	~60×	~76×
**Genome properties**		
Size* (bp)	86 772 227	53 163 602
N50	265 169	317 638
Number of contigs	1142	599
DNA G+C content (%)	51.7	51.6
Percentage coding	49.6	49.8
**Protein-coding genes**		
Number of gene models	27 522	17 371
Mean CDS length	1388	1484
DNA G+C content (%)	54.3	53.6
Gene density (genes per Mb)	320	326
**Intergenic regions**		
Mean length (bp)	1690	1660
DNA G+C content (%)	46.0	46.8
**RNA genes**		
tRNA	115	94
rRNA locus**	8	14
rRNA 5S	193	77
SL-RNA	206	622
snRNA	16	13
snoRNA	1561	1024

*Includes all contigs >5Kb.

**Includes SSU+5.8S+LSU.

A second noticeable improvement is related to the repetitive sequences clusters, which were collapsed in the previous assembly, and now are disaggregated into the actual copy number (Fig. 1). This is more clearly shown in Fig 1b that shows a magnification of a fragment from the same CL Brener chromosome, containing several clusters of tandem repeats. The figure also shows that some of these collapsed clusters contributed to assembly fragmentation, since they are located at contig boundaries.

A comparison between the same chromosome of TCC and Dm28c (Fig. 1c) revealed additional aspects concerning cluster repeats and structural variation between strains. Specifically, the figure shows copy number variations between the two strains in four groups (indicated by numbers 1 to 6 in Fig. 1c) as well as strain-specific insertions/deletions. Among the latter, it is worth mentioning a large segment located on the (left) telomere present in TCC and CL Brener, but absent in Dm28c, containing tandem repeats of some species-specific genes (such as DGF1).

Overall, in terms of integrity (i.e. low fragmentation levels) the assemblies obtained for both strains, represent notable improvements (Table 1) compared with previous ones. Their N50 values were 265 and 317 kb for TCC and Dm28c respectively, representing more than a ten-fold increase in this index. Furthermore, several contigs correspond to entire chromosomes (for instance contigs TCC_3, TCC_4, TCC_10, Dm28c_6, Dm28c_22 and Dm28c_58, being the largest ones, 1.65 Mb for Dm28c and 1.35 Mb for TCC). Other contigs are considerably smaller (50 kb) and are composed uniquely of a well-known satellite sequence of 195 bp that encompasses more than 5 % of the genome (see below). This extremely abundant repeat is one of the factors that contributes the most to assembly fragmentation. This occurred not only in previous assemblies with short reads, but its incidence is even significant in assemblies based on long reads, such as that presented here. Thus, this indicates that the size of some of its clusters exceeds that of the reads.

Besides fragmentation, another important element to consider when evaluating assembly quality is its size. In the case of Dm28c, the assembly size was 53.2 Mb, consistent with its haploid size as previously estimated using a fluorescent nucleic acid dye [50]. Previous efforts to sequence Dm28c and Sylvio X10/1 strains (two closely related TcI strains) using other technologies [6, 7] resulted not only in assembly fragmentation but also in genome sizes of roughly one half of the actual haploid size (27 Mb for Dm28c and 23 Mb for Sylvio X10/1). This size underestimation is in all likelihood due to the limitations of short-read technologies for assembling complex genomes. Keeping this limitation in mind, Franzén *et al.* [8] recalibrated their genome size estimation for Sylvio X10/1 to 44 Mb, extrapolating on the basis of non-assembled reads.

**Fig. 2.**
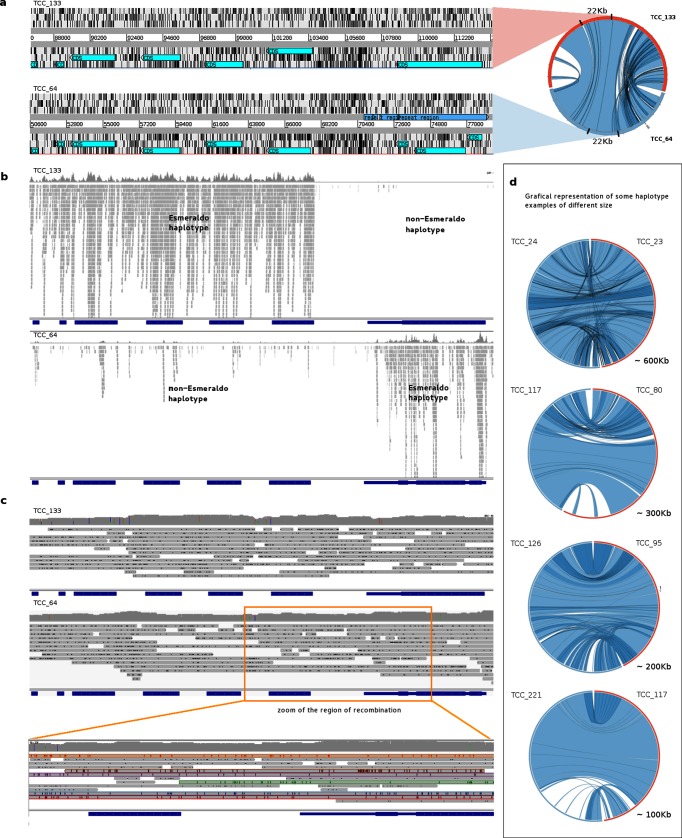
Haplotypes resolution and recombination. (a) Circos graph representation of homologous contigs (right). On the left is shown the Artemis view of the indicated fragments (for contig TCC_133 from 88 to 112 kb (top), and for contig TCC_64 from 50 to 77 kb (bottom)]. The six frames are shown and the annotated genes are represented in turquoise. (b) Alignment visualization (IGV) of the Esmeraldo Illumina reads (SRA833800) on the same homologous regions considered in (a) (TCC_133 on the top, TCC_64 on the bottom). (c) Alignment visualization (IGV) of PacBio TCC reads on the same region as in (b). On the bottom is represented the enlargement of the boxed region where Esmeraldo Illumina reads go from mapping to TCC_133 to mapping to TCC_64. (d) Circos graph representation of haplotype resolution contigs of different sizes.

The TCC strain is closely related to CL Brener, hence, we expected their genomes to be very similar in terms of size and sequence composition. Indeed, sequence identity is higher than 99.7 % over aligned segments longer than 10 kb (Table S1). The CL Brener genome has been estimated to be between 106 [3] and 122 Mb [50]. The assembly of TCC reported here consists of a ‘diploid’ genome of 86.7 Mb. However, the total assembly length is virtually the same if only contigs longer than 10 kb are considered (85.5 Mb). This value is below the expected genome (diploid) size, if one assumes that it should be similar to CL Brener, yet it represents a very significant improvement compared with the best previous assembly obtained by Weatherly *et al.* [9], in which both CL Brener haplotypes combined (Esmeraldo like and Non-Esmeraldo) have a total added size of only 54 Mb [9]. The smaller size of our assembly can be attributed to two factors. First, like CL Brener, TCC is a hybrid clone composed of two relatively divergent parental lineages similarly to Esmeraldo and non-Esmeraldo. This would imply that the distinction and ‘segregation’ of parental haplotypes was partial (about 40 % appears to have remained un-separated). It is likely that some genomic regions exhibit interallelic (inter-haplotype) divergence below the identity threshold that the assembler requires to discriminate between them. Secondly, it is possible that some clusters of repetitive sequences, especially the largest ones, were not completely uncollapsed, thus contributing to assembly shortening. It is difficult to estimate the effect of this second source of uncertainty on the assembly, since in the case of Dm28c, in spite of repetitions, the assembly was not smaller than the expected genome size. Importantly, however, for TCC we were able to distinguish and assemble separately (to a substantial degree) the parental haplotypes (Fig. 2).

### Separate assembly of parental haplotypes and recombination

The dissection of the genomes into their constituent haploid genomic blocks is important for studying some aspects of the evolutionary process that have been taking place in *T. cruzi* and could yield novel insights into the generation of hybrids and their subsequent evolution. An opportunity offered by our data, is the ability to detect events of homologous recombination between haplotypes. With this in mind, we mapped Illumina reads from Esmeraldo (TcII) (download from SRA: WUGSC SRX271443) to the TCC genome. The two haplotypes exhibit a divergence of 5.4 % on average (so reads of 75 nt in length are expected to have more than two mismatches with the non-Esmeraldo haplotype). This analysis was performed with high stringency (allowing no mismatches) so that reads would basically map only to the Esmeraldo-like haplotype. The separation was evidenced by the read mapping distribution: almost all of them mapped only to one of the homologous contigs (Fig. 2b). However, in sites where recombination presumably took place, there is a switch in the mapping pattern and reads start to map on the homolog contig, as shown in Fig. 2(b). To confirm that the switch in the mapping preference was not an assembly artifact; namely a chimera generated by mixing the two haplotypes, we mapped PacBio reads that span the region of recombination to these sites (Fig. 2c). As shown in Fig. 2(c), mismatches between reads and the contigs are evenly and randomly distributed along the genome. In contrast, when mapped into chimerical assembled contigs (Fig. S4), PacBio reads have low or high abundance of mismatches before and after the artificial ‘recombination’ point. The mapping pattern of Illumina reads onto chimerical contigs is, nevertheless, indistinguishable from that expected in actual recombination zones.

Another noteworthy observation from Fig. 2(b) is an extreme scarcity of Illumina reads mapping on this putative recombination hotspot. To investigate this we tested several possible causes. First, we wondered whether this could be the result of our stringent mapping conditions. However, relaxing mapping restrictions to allow more mismatches only caused a marginal increase in mapping. The same is true for 454 reads (from NCBI PRJNA50493); very few of them map here, even when a large percentage of mismatches is tolerated. To us, this indicated that this region is either refractory to sequencing or an artifact of our assembly. To test the latter, we searched for the presence of the region in other assemblies and strains. It was found in Dm28c (our assembly), Sylvio X10/1 (from NCBI PRJNA40815, PacBio) and CL Brener (Sanger). This led us to conclude that this region is poorly sequenced using other NGS technologies. Further analysis of the region, revealed a poor GC content, averaging 30 %, (with segments longer than 100 nt with GC content lower than 20 %). This is in keeping with previous reports showing that genomic segments with very low GC content are under-represented in Illumina sequencing [51]. Using GC content and Illumina sequencing depth as criteria for identifying similar regions in the genome of TCC, we found more than 500 regions simultaneously matching these two features: very low GC content and very few or no mapping reads (with segments where sequencing depth is as low as zero). A similar search in Dm28c (PacBio assembly), yielded 160 such regions. Comparing with other assemblies we found that most GC-low regions from TCC are also present in CL Brener assembly, but less than half of them can be found in our assembly of Dm28c. The comparison of two Dm28c assemblies reveals that only 90 of the 160 found in the PacBio assembly, reported here, were present in the Dm28c assembly obtained using Roche 454 reads. Similar figures are obtained when the comparison is performed with Sylvio X10/1 (Illumina and Roche 454). Furthermore, most of these regions, or fragments of them, are located in contigs ends (for Illumina and Roche 454) indicating that assembly was halted at these positions due to insufficient coverage. Taken together, these results indicate that these low-GC regions are another important factor in assembly fragmentation in *T. cruzi* genomes for Illumina and 454 technologies, but they do not appear to affect Sanger- or PacBio-based assemblies.

### Genome annotation

Genome annotation is an error-prone task, which is particularly intricate in *T. cruzi* due to its intrinsic genomic complexities, including large multigene families, pseudogenes and the absence of fully annotated genomes from phylogenetically closely related species. To address these problems, we followed an annotation strategy designed to handle the peculiarities of this species. Since the vast majority of trypanosome genes lack introns, we used a ‘prokaryotic-like’ approach to identify them. Specifically, as gene models to be used downstream in the annotation pipeline we used a greedy criterion: all open reading frames (ORFs) longer than 450 nt and starting with Met. Shorter CDSs were dealt with separately (see below). Since many long ORFs are not protein-coding, this initial group of potential protein-coding genes very probably contains a large number of false positives. However, only those ORFs encoding proteins will yield blastp hits with relatively distant species, hence, these can be readily identified and excluded by subsequent filtering with blastp, using appropriate protein reference data sets. Therefore these data sets are crucial to get accurate annotations. Indeed a common drawback in genome annotation is inheriting (by homology transfer of information) spurious and erroneous annotations from other genomes used as references. To work around this potential source of inaccuracy the following datasets were built/used: (i) A carefully curated databases of *T. cruzi* multigene families (TS, mucin, MASP, GP63, TASV, DGF-1, RHS). Each of these families was thoroughly scrutinized manually to determine the complete and probable functional copies, their sub-groups and pseudogenes. (ii) Trypanosomatid proteome databases from which *T. cruzi* strains as well as other species not very distant from *T. cruzi* (*T. rangeli*, *T. grayi*) were excluded. The rationale for this criterion is that whereas evolutionary conservation is an indication of functional relevance (i.e. if the amino acid sequence encoded by the ORF has been maintained over time this indicates a real protein-coding gene), conservation among not distant taxa does not guarantee functional relevance; instead, it could merely reflect phylogenetic inertia (i.e. there not being enough time to diverge). (iii) A curated protein database from non-trypanosomatids (iv) Additionally, to cross-check our annotations, we used protein annotations from *T. cruzi,* exclusively from CL Brener, since this is the strain most accurately annotated. Whenever inconsistencies arose, problematic ORFs were further analyzed and manually curated. An overview of the workflow is presented in Fig. S1.

In order to include, and annotate, protein-coding genes shorter than 450 nt, the search was conducted backwards; namely a dataset of short proteins was built and used to search for them (using tblastn) in the assemblies. Diverse tools (see methods) were used to incorporate additional information in the annotation. Non-coding RNAs, transposable elements and repetitive elements, were annotated using dedicated software run on specific databases that were built for these purposes.

Although this strategy is intended to minimize misannotations, further manual curation was needed, especially with genes belonging to multigene families and their associated pseudogenes. In particular, several of these genes had been frequently excluded from previous assemblies, as they were impossible to accurately position [9]. The genomes of the two strains presented here contain 27 522 and 17 371 protein coding genes (TCC and Dm28c respectively) and the number of hypothetical genes has been reduced from ~50 to ~39 % by means of the removal of spurious annotation due to the inclusion of new gene functions that could be classified due to the improvements of the TriTrypDB, or because they were coding sequences that were not assembled before.

To visualize and comfortably handle the information on the *T. cruzi* genomes we generated a web platform (http://bioinformatica.fcien.edu.uy). A genome browser can be used to navigate the annotation of contigs longer than 50 kb. It also offers built in tools to select groups of genes and other genomic features that are distinctive for *T. cruzi*, such as surface multigene families, repetitive elements and directional gene clusters. Among other functionalities of the interface, it is possible to visualize gene annotations and retrieve their nucleotide and amino acid sequences. It is also possible to conduct different searches (by annotation or by keywords) and to visualize graphical representation of repetitions and haplotypes (haplotype information is available). The interface is intuitive and user friendly. See Methods for further details.

### The genome of *T. cruzi* is compartmentalized

**Fig. 3.**
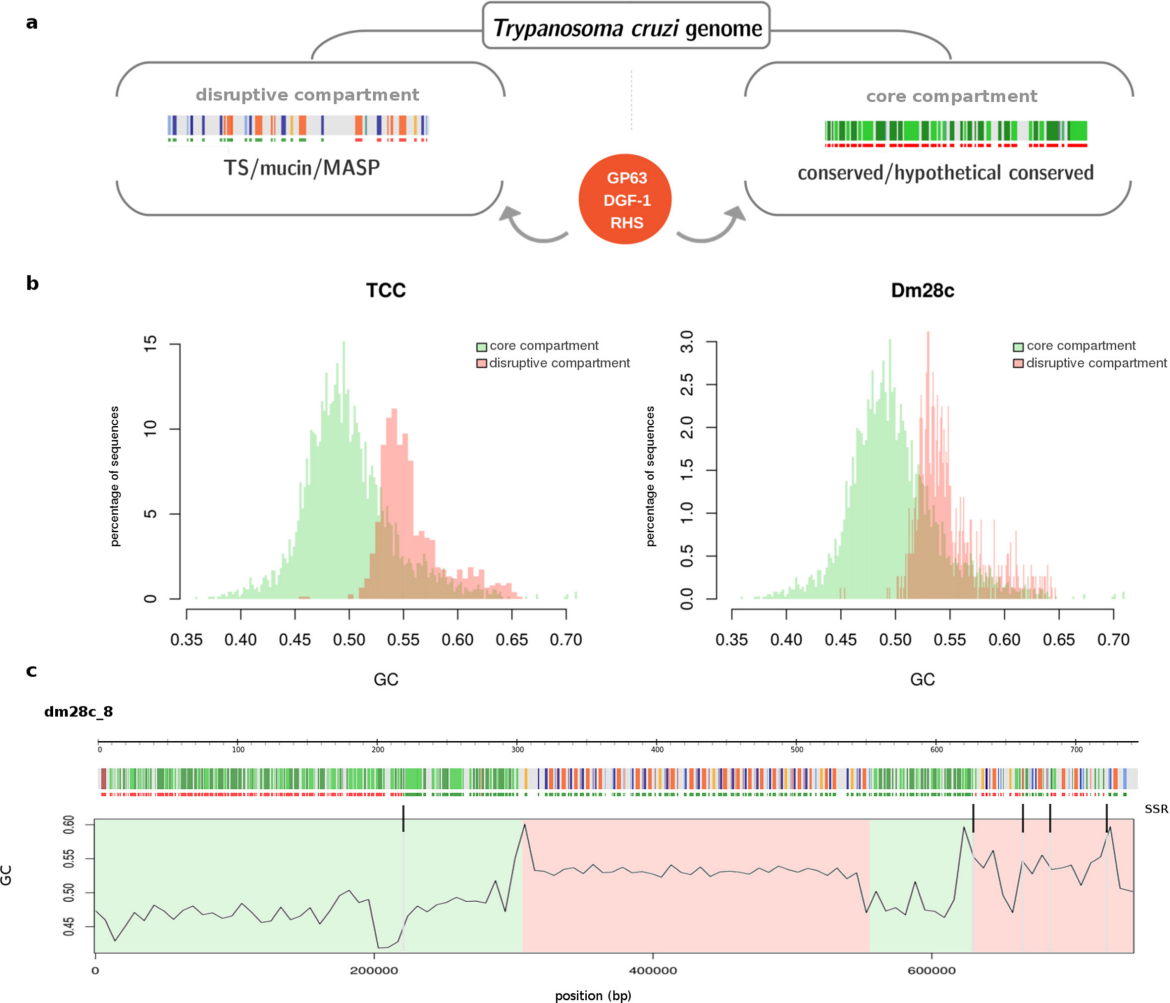
The genome compartmentalization of *T. cruzi*. (a) Schematic representation of the two types of compartment in *T. cruzi*. Genes are visualized as in the web interface by strips (DGF1 red, GP63 orange, MASP blue, mucin light blue, TS light orange, conserved genes green). The core compartment is composed of conserved genes. The disruptive compartment is composed of surface multigene families TS, MASP and mucins. GP63, DGF-1 and RHS are distributed (sometimes in tandem clusters) in both compartments. (b) GC distribution of the compartments. Only contigs entirely composed of one compartment (80 % or higher proportion of conserved genes or surface multigene families) and longer than 10 kb were considered. (c) Schematic representation of a contig of Dm28c; genes are depicted as in (a) and colour compartment as in (b). The GC distribution is calculated over a sliding windows of 7000 bp. Strand-switch regions are indicated above the GC plot by black vertical stripes.

Two groups of protein-coding genes were very well described in *T. cruzi*: the multigene families with hundreds of copies: DGF-1, GP63, MASP, mucins, RHS and TS, and those generically defined as ‘conserved’, which can be further divided into two groups: genes encoding proteins with a known function called ‘conserved genes’, and genes without an assigned function but present in more than one trypanosomatid species called ‘hypothetical conserved genes’. We found that the genome of *T. cruzi* is compartmentalized in two clearly defined regions: a ‘core compartment’ composed of conserved and hypothetical conserved genes, and a ‘non-syntenic’ ‘disruptive compartment’ composed of the multigene families TS, MASP and mucins (Fig. 3a). On the other hand, GP63, DGF-1 and RHS multigene families have a dispersed distribution in the genome, being present in both compartments, which may in turn be organized as unique or in tandem array distribution. Since the ‘core compartment’ corresponds to the previously described syntenic blocks in *T. brucei* and *L. major* [4], and the ‘disruptive compartment’ is mainly composed of species- or genus-specific genes, the latter can be considered as a recent region of the genome. In turn, all of the ncRNAs are located in the core compartment (see web interface, e.g. contigs TCC:1,TCC_9, TCC_57 and Dm28c_2 Dm28c_30). It is noteworthy that the members of the disruptive compartment have been previously referred to as sub-telomeric; however, as is evident from our results, these genes can be located in any position in the chromosome with the only condition that they cover wide ranges of distances, and the location can be anything from internal chromosomal regions to extremes of chromosomes, and even comprise whole chromosomes. Therefore the term sub-telomeric, probably inherited from the genome organization of VSG genes in *T. brucei*, is inappropriate and can lead to confusion. We suggest it should be replaced by the more encompassing concept of ‘compartments’.

In order to identify general features distinguishing the compartments, we performed compositional analysis of the genome, and found that the disruptive compartment consistently exhibits higher GC content than the core compartment (Fig. 3b). Interestingly, strand-switch regions show GC peaks that indicate particular compositions as shown in Fig. 3(c). In this figure is shown the GC distribution over the length of a contig, and the bias in GC content becomes clear (Fig. 3c). This genome organization resembles isochore-like structures. In fact, although originally only described in vertebrates [52, 53], the ‘isochoric’ level of genome organization was recently proposed to be a feature of all eukaryotes including unicellular ones [54, 55]. Our observations are consistent with this concept. This distribution deserves further study to examine how compartmentalization emerged and evolved.

### Multigene families

One of the known characteristics of the *T. cruzi* genome is its widely expanded content, mainly due to the large number of multigene families [35, 56, 57]. In fact, when the first genome of *T. cruzi* was sequenced, authors pointed out the risk of underestimating the number of units of genes organized in tandem [3]. Later, Arner and collaborators exposed examples of copy number underestimation and misassembly and proposed that the number of protein-coding genes and pseudogenes may be twice the previous estimates [58]. Our genome assembly makes it possible to determine the real extent of the *T. cruzi* gene expansion. Defining a gene family as that which presents eight or more genes, we identified 90 and 74 families in TCC and Dm28c, respectively. A more detailed analysis of families by MCL clustering indicated that they can be subdivided; TCC presents 190 paralogous gene clusters and Dm28c presents 139 (Table S2). In Table 2 we show only those gene families with more than 50 members in at least one sequenced genome. As previously described, the most enriched ones are TS, MASP, RHS, mucins, DGF-1 and GP63 [3]. Remarkably, whereas RHS, DGF-1, GP63 and TS fall into six or fewer clusters in both strains, mucins, and particularly MASPs, harbor higher number of subfamilies (clusters) (Tables 2 and S2). On the other hand, misannotation of mucins and TS led us to a more in detail analysis (see below). Interestingly, a more accurate estimation of copy number, and the analysis of the core compartment, allowed us to identify more than 20 families with at least 50 genes, including several genes defined as ‘housekeeping’ in eukaryotes, such as Hsp70 and Histones H2B and H3 (Tables 2 and S2). Given that gene expression is regulated mainly at the post-transcriptional level in trypanosomatids, the increase in the number of genes has been proposed as a mechanism for increasing gene expression level. However, by gene ontology analysis we could not find any particularly enriched metabolic pathway which could further strengthen this hypothesis from a functional perspective.

**Table 2.** Gene families groups in *T. cruzi*. The total numbers of genes and clusters are listed

	TCC*		DM28c*	
**Gene Product**	**Members**	**MCL clusters**	**Members**	**MCL clusters**
Trans-sialidase (TS)	1734	2	1491	3
MASP	1332	44	1045	38
RHS	1264	4	774	2
Mucins	970	21	571	10
GP63	718	6	378	6
DGF-1	491	1	215	1
UDP-Gal or UDP-GlcNAc-dependent glycosyltransferase	128	1	110	1
Protein kinase	152	6	118	4
Amino acid permease/transporter	128	1	93	1
Elongation factor (1-alpha, 1-gamma and 2)	109	3	63	3
Protein Associated with Differentiation	98	1	69	1
Glutamamyl carboxypeptidase	96	1	80	1
Syntaxin binding protein	91	2	40	2
TASV	87	5	53	3
Heat shock protein 70	87	2	40	1
Kinesin	85	3	55	3
Glycine dehydrogenase	73	2	51	1
Beta galactofuranosyl glycosyltransferase	73	1	37	1
Receptor-type adenylate cyclase	63	1	27	1
Histone H2B	56	1	5	1
Histone H3	54	1	27	1
Oligosaccharyl transferase	52	1	3	1
Tryptophanyl-tRNA synthetase	52	1	36	1
ATP-dependent DEAD/H RNA helicase	59	3	39	3
Casein kinase	50	1	42	1
Cysteine proteinase	46	1	56	1
Flagellar calcium-binding 24 kDa protein	41	1	52	1

*Only clusters with at least 50 members are shown.

This analysis also revealed specific clusters of hypothetical protein coding genes. We identified 47 (TCC) and 27 (Dm28c) novel multigene families of at least eight paralogues, with ten of these clusters containing 20 or more paralogues on each genome (Table S3). Among them we found shared and strain-specific families containing conserved domains (Table S3). Remarkably, the largest family is composed of 158 members in TCC (cluster 35, Table S3) meaning that, after excluding the multigene superfamilies, it represents one of the most enriched genes in the whole of the TCC genome (Table 2), indicating a relevant role in the parasite that deserves further study.

**Fig. 4.**
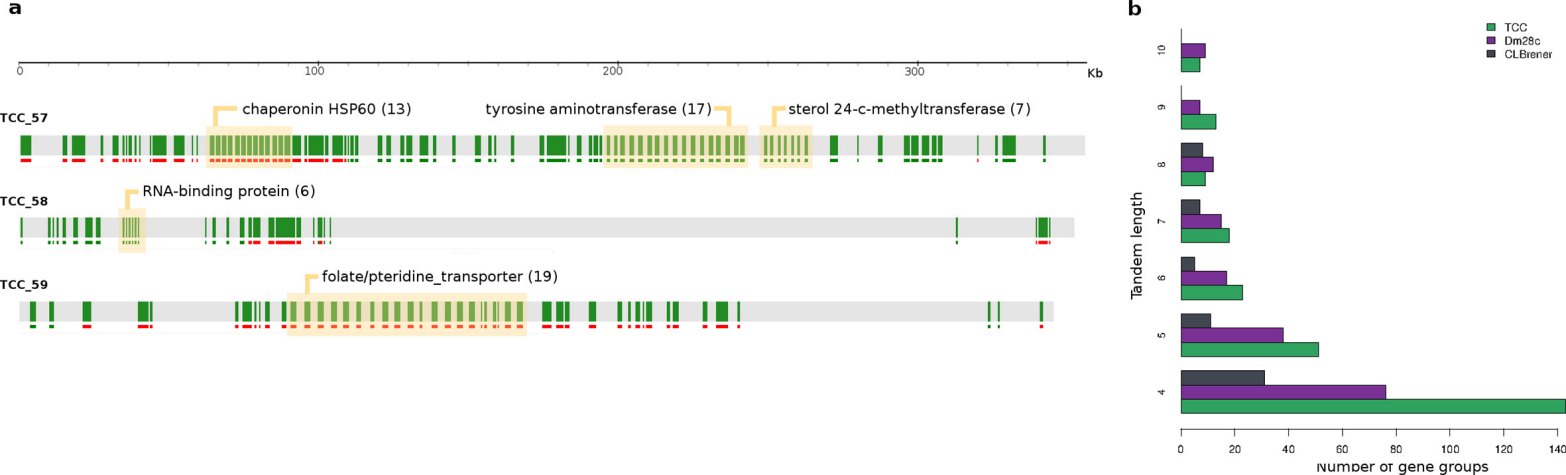
Tandem gene organization. (a) Representation of three contigs of TCC as in the web interface where only conserved genes are shown (green strips). Groups of tandemly arrayed genes are highlighted; parentheses indicate the number of copies. (b) Graph representation of the number of groups of tandemly arrayed genes (represented tandem length from four to ten genes) in the different genome assemblies. TCC in green, Dm28c in violet, CL Brener in gray.

Interestingly, clusterizations include all the genes from a family, both in tandem and dispersed. In fact, although tandems of genes have been already described in *T. cruzi*, the resolution of previously collapsed repetitive regions allowed us to visualize and measure the extent of the tandem arrays of genes; we identified 2363 (TCC) and 1619 (Dm28c) genes organized in tandems of at least two genes (excluding hypothetical genes). This organization, which was previously difficult to identify, is now evident by simply looking at the chromosomes (Fig. 4a and Web interface). Needless to say that tandem genes had been grossly underestimated in previous assemblies. Fig. 4(b) shows the increment of the number of tandem arrayed groups of genes, in comparison with the original CL Brener genome assembly; there are five times more gene tandems of four genes in TCC than those identified previously. One may wonder whether these differences between the CL Brener and TCC assemblies described above could be attributed to real genomic differences between the two strains. We consider this possibility as very unlikely because they are precisely the kind of differences one would expect in assemblies obtained using these two technologies. Additionally, it is worth taking into account that TCC and CL Brener are almost identical at the sequence level (identity higher than 99.7 %). It would constitute an unprecedented situation having such a big difference in copy number without exhibiting DNA sequence divergence.

### Single-copy genes

Although multigene families encoding surface proteins are the main cause of genome expansion, this feature is a more general phenomena of *T. cruzi*, affecting the majority of its genome content. In fact, most genes have at least two copies per haploid genome. Accuracy in gene copy number determination is highly relevant for functional studies, since knock-out experiments and the generation of transgenic strains constitute an essential source of information to determine whether a gene is essential, paramount to defining new drug targets for treatment of Chagas disease. Keeping this in mind, we wondered what the actual core of single-copy genes was. We found approximately 5000 single-copy genes in Dm28c, of which only 1377 have an assigned function. We then focused our analysis on some genes for which knock-out experiments had been reported (Table S4). First we noticed that a number of genes previously considered to be single-copy form a cluster of a few copies, which led to miscalculations when semi-quantitative methods were used. Such is the case for lipoamide dehydrogenase, for which the copy number had been determined by Southern blot [59]; this gene exhibits seven copies in TCC and six in Dm28c. *msh2* [60] has four and two copies in TCC and Dm28c, respectively, and *hgprt* [61] presents four copies on each genome. *T. cruzi* has been traditionally considered an organism refractory to genetic manipulation. The difficulties in performing gene knock-out (e.g. to insert genes of resistance to a drug with 5′ and 3′ flanking regions of the target gene) has been ascribed to particular features of its recombination machinery. However, further analysis of some successful knock-out experiments reported [62] revealed that all of the genes involved (*gp72, lyt-1, kap3*) exhibit unique copies in both strains [63–65]. We propose that copy number underestimation could account for the failure of classical knock-out experiments in this parasite. Conversely, single-copy genes that could not be completely knocked out, such as calreticulin, *dhfr-ts* and *cub*, but for which instead monoallelic deletions were obtained should be considered essential [66–68]. Thus, long-read sequencing allowed correct estimation of gene copy number, leading us to reconsider the assumption that classical gene knock-out methods do not work in *T. cruzi*. On the other hand, our data can also aid in predicting the feasibility of mutagenesis experiments done using CRISPR technology whereby the higher the copy number, the higher the difficulty of ‘hitting’ all copies simultaneously.

### Mucins and trans-sialidases

The biological relevance of mucins and trans-sialidases in infection, in addition to the high number of incomplete genes and imprecise pseudogene number determination, motivated us to manually curate these genes.

Mucin-like proteins of *T. cruzi* were classified into three main groups, TcMUCI, TcMUCII and large and small TcSMUG (TcSMUGL and TcSMUGS respectively). TcMUCI and TcMUCII are expressed in the mammalian stages of the parasite, having a highly variable region and molecular weights ranging from 80 to 200 kDa [35, 69], whereas SMUG genes, smaller than TcMUC and with little variability, are mainly expressed in the insect-derived stages [57, 70–72]. Initially classified as TcMUCI and TcMUCII, the presence of a mosaic of sequences intermediate between the two groups led to the proposal of a common ancestor and further diversification [35]. In this work we considered as complete mucin genes those whose deduced amino acid sequences had an N-terminal SP, a C-terminal GPI anchor sequence and T-rich sequences such as T8KP2, T6-8KAP or T6-8QAP. With these criteria and including RNA-seq data, we performed manual searches, including correction of initial methionine in some cases. In this manner, we identified a total of 970 and 571 mucin genes in TCC and Dm28c, respectively, of which 247 (TCC) and 113 (Dm28c) corresponded to pseudogenes (Table 3). Comparison between the two strains led to practically the same profile of mucin groups; around 60 % were classified as TcMUCII and only 6 % as TcMUCI. However, we found differences in the distribution of the TcSMUG family. In TCC approximately 6 % were TcSMUGS and approximately 3 % were TcSMUGL, while the opposite occurs in Dm28c, where approximately 4 % were TcSMUGS and approximately 10 % were TcSMUGL. We found 56 genes coding for TcSMUGL in Dm28c and only 25 in TCC. These findings raise the interesting possibility that these differences may be associated with phenotypic differences in virulence. Indeed, it has been shown that TcSMUGL products contribute to *T. cruzi* infectivity towards the insect vector [73]. On the other hand they could be only related to DTUs polymorphisms, thus this issue deserves further investigation on different strains of *T. cruzi*. Our analysis also revealed the presence of a large number of mucin fragments and/or pseudogenes, in a similar percentage for both strains: 25 and 20 % in TCC and Dm28c, respectively.

**Table 3.** Manually annotated surface multigene families

	**TCC**			**Dm28c**		
	Total	Genes	Pseudogenes	Total	Genes	Pseudogenes
TS	1734	689	1045	1491	709	782
MASP	1332	941	391	1045	736	309
GP63	718	237	481	378	96	282
DGF-1	491	191	300	215	75	140
Mucin	970	723	247	571	458	113

**Mucin**	**Total**	**TcMUCII**	**TcMUCI**	**TcSMUGS**	**TcSMUGL**	**Pseudogenes**
TCC	970 (100%)	585 (60.3%)	57 (5.9%)	56 (5.8%)	25 (2.6%)	247 (25.5%)
Dm28c	571 (100%)	351 (61.5%)	29 (5.1%)	22 (3.9%)	56 (9.8%)	113 (19.8%)

Trans-sialidase/trans-sialidase-like proteins constitute a large and polymorphic superfamily of around 1400 members. This heterogeneous family is currently classified in eight different groups [37], and although most of them do not exhibit trans-sialidase activity they play relevant roles in infectivity and virulence [37]. TS contain an N-terminal SP and a C-terminal GPI anchor signal. Functional members of the TS family possess the characteristic VTVxNVLLYNR motif. Additionally, some groups have N-terminal ASP box sequences. Members of Groups I and IV also contain SAPA repeats, an extremely antigenic region whose role is to increase the half-life of TS in blood [37, 74]. The initial annotated TS sequences were manually filtered using the following criteria: presence of VTV and ASP motifs, GPI anchor signal probability and presence of a SP sequence. Using these criteria we found 1734 TS genes in TCC and 1491 genes in Dm28c. However, using the same methodology as Freitas *et al*. [37], we could not identify the eight groups described for TS, indicating that this classification is not robust enough or might be species-specific. Our analysis also revealed the presence of a large number of pseudogenes or fragments; 721 and 567 for TCC and Dm28c, respectively (Table 3). These results highlight the importance of manual curation for the most expanded and complex multigene families. Finally, the fact that TS genes, present also in *T. brucei*, were found in the disruptive compartment deserves future analysis, in order to evaluate how the more ancestral genes are distributed and to obtain clues about the evolution of this family.

### Transposable elements

Transposable elements (TEs) are dynamic drivers of evolutionary processes that contribute to genomic plasticity. Usually present as repetitive sequences in the *T. cruzi* genome, they are abundant and commonly misannotated. *T. cruzi* presents three families of autonomous genomic elements, as well as their non-autonomous pairs. Here, we were able to identify the entire sequences of all TEs families present in both genomes and their flanking sequences (Table 4). Namely, VIPER, a tyrosine recombinase (YR) element which belongs to the DIRS order; L1Tc, a non-LTR element of the INGI clade; and CZAR, also a non-LTR element from the CRE clade which is site-specific inserting only in the SL gene [3, 75–77]. On the other hand, non-autonomous elements have also been identified. Namely SIDER, which has sequence similarity to VIPER´s 5′ and 3′ ends; NARTc, the non-autonomous pair of L1Tc elements; and TcTREZO [78]. Putative active and defective copies could be detected for all families. None of the VIPER, CZAR and TcTREZO elements had complete domains, indicating that all copies are defective, whereas L1Tc was the only one to show putative active copies in both genomes.

**Table 4.** Complete retrotransposon copy numbers in *T. cruzi*

**Retrotransposons**	**TCC**				**Dm28c**			
	Complete copies	Length (kb)	Identity (%)	GC content (%)	Complete copies	Length (kb)	Identity (%)	GC content (%)
Non-LTR retrotransposons								
CZAR	43	6.497	93.2	55.7	57	6.442	91.9	56.3
L1Tc	43(13)	4.874	90.7	53.0	54(18)	4.749	96.5	53.3
NARTc*	110	0.257	92.6	51.7	55	0.258	90.5	51.7
TcTREZO	978	1.459	92.1	50.9	297	1.423	95.2	50.3
YR retrotransposons								
VIPER	244	3.454	85.0	55.2	194	3.423	87.3	54.8
SIRE*	851	0.440	87.4	44.0	669	0.441	88.6	44.2

*Non autonomous.

Parentheses indicate the number of putative active copies.

**Fig. 5.**
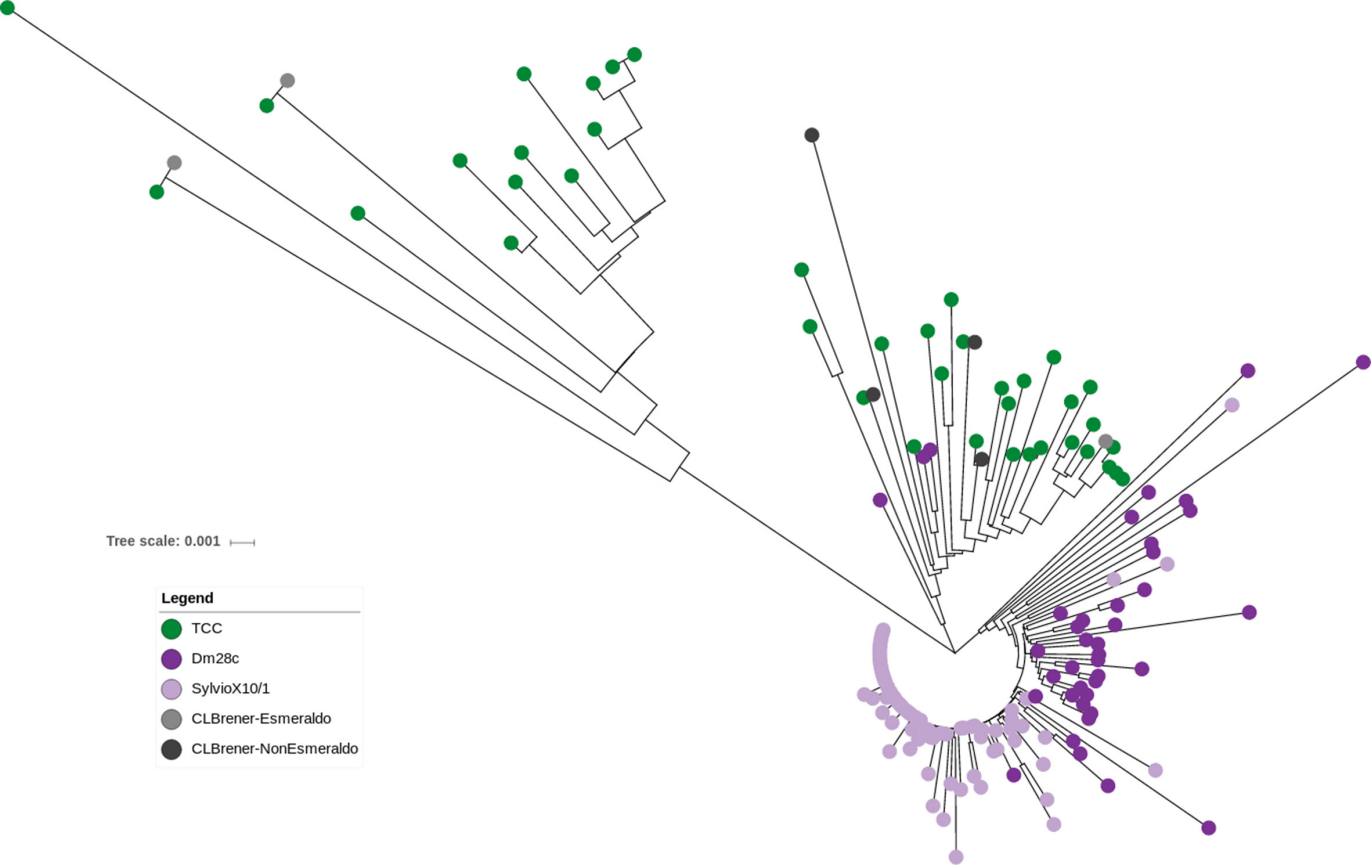
L1Tc phylogeny. Maximum-likelihood phylogeny of complete sequences of L1Tc. Elements from TCC in green, Dm28c in violet, SylvioX10/1 in light violet, CL Brener Esmeraldo-like in light violet grey, CL Brener non-Esmeraldo-like in b.

To shed some light on the evolutionary dynamics of L1Tc transposon, we first built a maximum likelihood phylogenetic tree using only complete, and hence putatively functional sequences of this element. The resulting tree (Fig. 5), exhibits three main clades, one containing L1Tc copies from Dm28c, and two clades containing TCC copies. To further explore this configuration, we analyzed (full length) L1Tc elements from CL Brener, discriminating Esmeraldo and non-Esmeraldo haplotypes. It becomes immediately apparent that while one TCC cluster is associated with the Esmeraldo haplotype, the second TCC cluster is related to the non-Esmeraldo haplotype (Fig. 5). This provides evidence that the functional copies of L1Tc had significant activity and underwent substantial evolutionary divergence after the two ancestral *T. cruzi* lineages comprising TCC (and CL Brener) split apart. The fact that branches in these two clusters are considerably longer, denotes a low transposition/divergence ratio. It is surprising, however, that there are hardly any intermingled copies, meaning that any inter-copy variability predating the separation of these two ancestral lineages (i.e. already present in the ancestral genome) has been erased. This is consistent with the fact that Dm28c copies are also isolated, forming a single cluster. Nevertheless, in this case most copies are very similar to each other (short branches) indicating that in this strain, L1Tc exhibits a much higher transposition : divergence ratio. One can attribute this to the fact that the new copies did not have enough time to diverge after their emergence owing to a high transposition rate. Alternatively, this might be due to a much slower nucleotide evolutionary (divergence) rate. Incorporating in the analysis the set of L1Tc sequences from the strain Sylvio X10/1 (from NCBI PRJNA40815) gives some clues as to the cause of the different dynamics. In effect, these sequences cluster together with those of Dm28c, yet a sharp division between the two strains is observed (Fig. 5). Since the two strains are very close relatives (with virtually identical nucleotide sequences genome-wide), short branches in this part of the tree cannot be attributed to a slow divergence rate, favoring the hypothesis of very high transposition rate in this DTU. In any case, it denotes a markedly different dynamic from that observed in the hybrid TcVI. Whether the different dynamics are related to the hybrid/non-hybrid nature of the strains being considered is a subject that deserves further assessment.

### Tandem repeats

Tandem repeats (TR) are considered ‘neglected’ sequences in genome analyses, since short reads cause often unsolvable problems for *de novo* assemblies in TR regions. As a consequence of long reads we could resolve TR-enriched regions previously fragmented. As expected, thousands of copies of the already well characterized 195nt satellite [79, 80] were found: 41 061 (8.3Mb) and 12 244 (2.5Mb) copies for TCC and Dm28c, respectively (Table 5). These findings are in keeping with previous estimates made by cell and molecular biology methods [78, 81]. The difference in copy number between the genomes analyzed are not a consequence of the different genome sizes only. In fact, it has been observed that satellite amounts are variable among strains, reaching differences of up to sixfold, but organized similarly throughout the genome [81]. The results of our analysis indicate that the satellite is two times more abundant in TCC than in Dm28c, accounting for 9.5 and 4.7 % of the total size of the genomes, respectively (Table 5). This coincides with the differences estimated between TcVI and TcI groups by Souza *et al.* [*7*^8^], the repeats in the former being two to four times more abundant than in the latter. As mentioned, the abundance of the satellite led to a high level of assembly fragmentation. In this work we recovered satellites reaching lengths of up to 45 and 42 kb in TCC and Dm28c, respectively, although the majority were of smaller size, with an average of more than 10 kb, as previously reported [81]. We could assemble several contigs containing satellite sequences surrounded by single-copy DNA, meaning that in these cases it was possible to pass over these repetitive clusters (Fig. S5). However, the contribution of this satellite to fragmentation is notorious, since some satellite sequences are located at the end of many small and medium-sized contigs (see for example TCC_301, 303, 312, and Dm28c_122, 126, 146).

**Table 5.** Tandem repeats in TCC and Dm28c

**A**	**Satellite**	**Number of tandems**	**Total length (bp)**	**Percentage of genome**	**Mean identity (%)**
TCC	195 bp	41 062	8 303 881	9.5	95.4
Dm28c	195 bp	12 244	2 483 120	4.7	95.1

**B**	**Tandem length**	**Number of groups**	**Total length (bp)**	**Mean identity (%)**	**Belong to or include genes (%)**
**TCC**	3 to 10	74	154 516	85.5	39.2
	11 to 100	135	833 791	84.2	74.1
	101 to 500	26	343 243	94.7	80.8
	501 to 2000	26	1 528 929	97.6	92.3
**Dm28c**	3 to 10	59	117 028	85.0	37.3
	11 to 100	104	185 816	84.1	62.5
	101 to 500	18	301 938	96.0	66.7
	501 to 2000	24	1 091 688	97.9	91.7

We then aimed to characterize the remaining TR sequences with a minimal length of three nucleotides and containing at least ten monomers. We identified 261 and 205 groups of different sizes the longest period in both genomes being approximately 1960 bp (Table 5).

The identified TR (related or not to coding sequences) described here are shared between the strains analyzed. Around 85 % of TR are present in both genomes, although only 107 groups of TR are shared. This means that the remainder, even when they are present in the other genome, do not meet the selection criteria; they do not have at least ten copies or enough identity between copies (*i.e.* they are more degenerate in one genome than in the other).

It is noteworthy that several TR are composed of protein-coding genes organized in tandems where the intergenic regions are highly conserved. Such is the case of Flagellar Attachment Zone protein 1, R27-2 protein, Kinesin-like protein and histone H4 among others (Table S5). This could represent recent events of consecutive gene duplication that, in contrast with the tandem arrays of protein-coding genes described above, still retain high levels of identity among their intergenic regions. In fact, around 90 % of the TR with periods from 500 to 2000 bp correspond to these arrays, and the tandem intra-identity is above 97.5 % (Table 5). Conversely, we detected TR of shorter periods present within coding sequences (Table 5). These internal tandem repeats are multiples of three, which indicates that they will be translated as a tandem of amino acids. This feature was first identified on the shed antigen SAPA [82], and subsequently, by immunoscreening of expression libraries [83, 84]. Additionally, TRs have been observed in TS, mucins and MASP, and in several other hypothetical proteins exhibiting signal sequences or transmembrane domains, some of which have been determined to be antigenic [85]. Antigenicity of these tandems, and their wide distribution along CDS indicate that they constitute a general mechanism for including unspecific polyclonal immune responses. These could act as ‘smoke screens’, which could allow the parasite to evade the host immune response.

## Conclusions

In this work we present the assembled and annotated genomes of two *T. cruzi* clones: the hybrid TCC and the non-hybrid Dm28c obtained using PacBio long-read technology. This allowed us to overcome, to a great extent, the common complications associated with this genome: assembly fragmentation and collapse of repetitive sequences. The improved assemblies obtained herein permitted us not only to accurately estimate copy numbers of tandemly arrayed genes and multigene families, but also to unambiguously identify many single-copy genes. We also found that the genome of *T. cruzi* is composed of a ‘core compartment’ and a ‘disruptive compartment’ which exhibit significant differences in DNA GC content and gene composition, the former being GC-poorer and composed of conserved genes, and the latter enriched in TS, mucins and MASP genes. In addition, many homologous chromosomes (haplotypes) were separately assembled, and some homologous recombination events could be identified. Finally, manual annotation of surface multigene families, mucins and trans-sialidases allows now a better overview of these complex groups of genes. These genomes and their annotation can be visualized and navigated using a customized web interface created in this work (http://bioinformatica.fcien.edu.uy/) and are also available in public repositories (NCBI and TriTrypDB).

## Supplementary Data

886 Supplementary File 1

## Supplementary Data

116 Supplementary File 2

## Supplementary Data

33 Supplementary File 3

## Supplementary Data

24 Supplementary File 4

## Supplementary Data

7 Supplementary File 5

## Supplementary Data

144 Supplementary File 6
