# Supplementary material for: Expanding an expanded genome: long-read sequencing of Trypanosoma cruzi
Source: Microb Genom. 2018 Apr 30;4(5):e000177. doi: 10.1099/mgen.0.000177 (PMC5994713; doi:10.1099/mgen.0.000177)
Supplement: Supplementary File 1 [file mgen-4-177-s001.pdf]

Main annotation pipeline

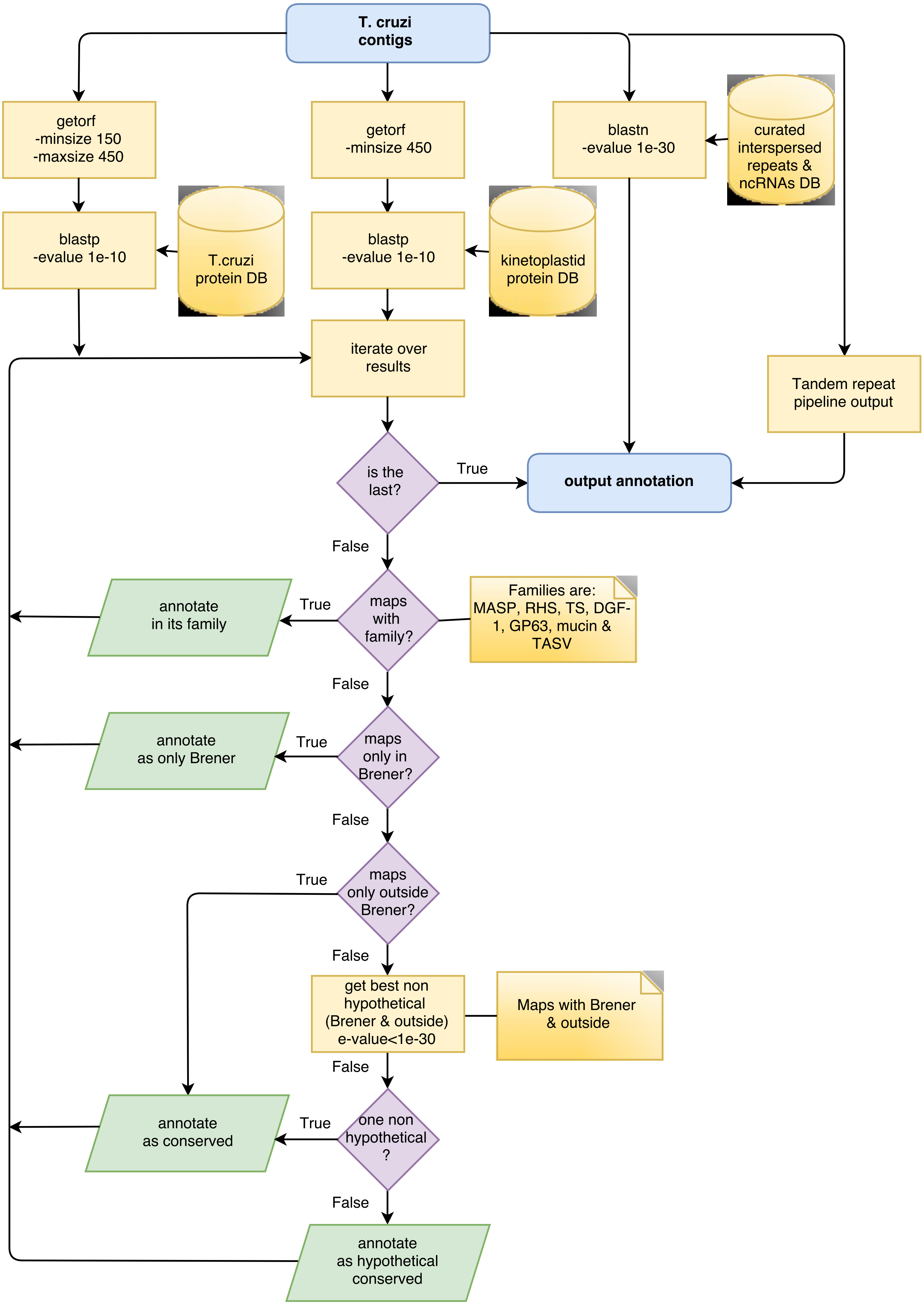

Fig. S1. Annotation workflow. Illustration of the main steps performed to generate the annotation of both genomes. Database and parameters used are explained. See Methods for more details.

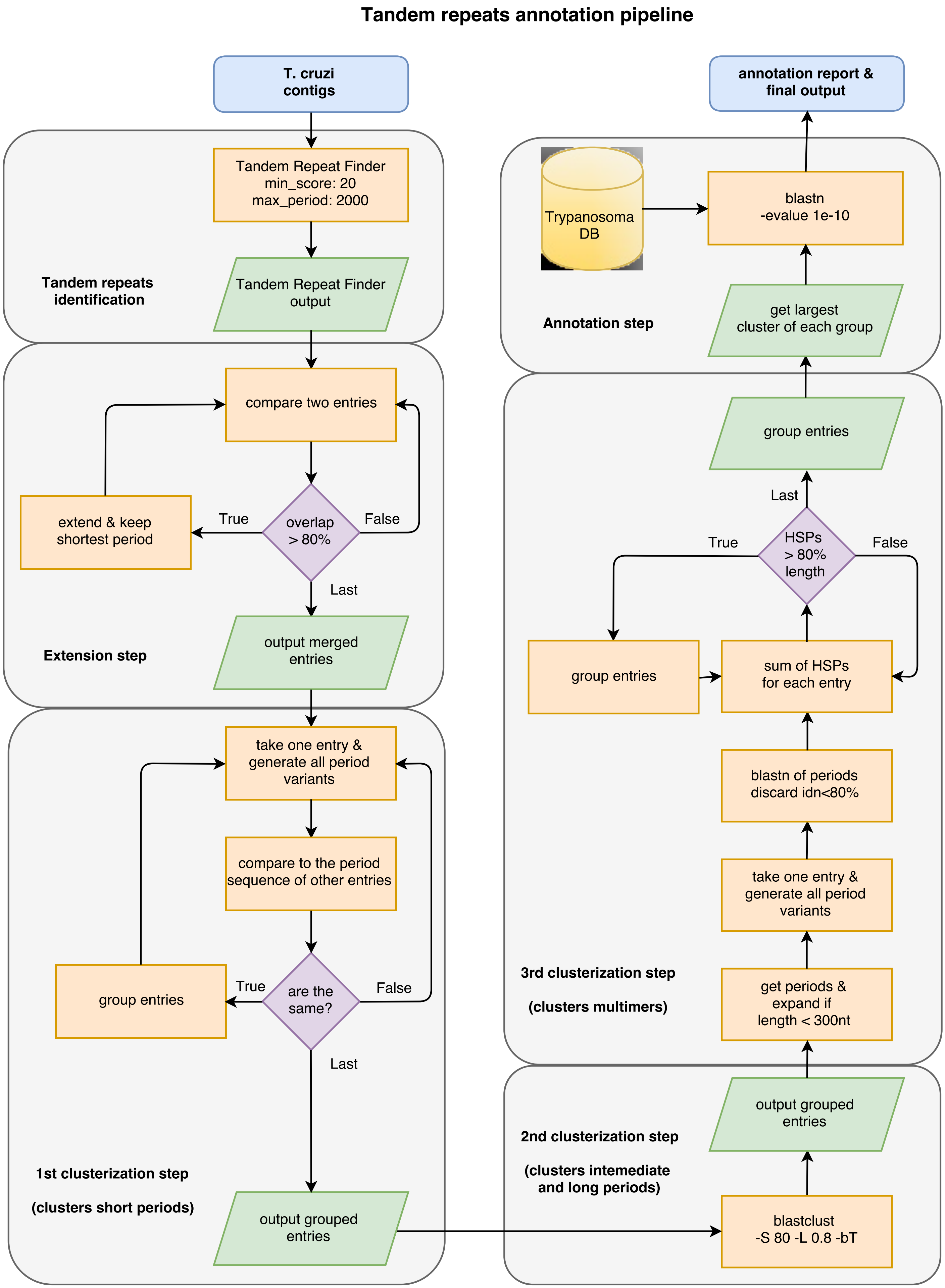

Fig. S2. Tandem repeats annotation pipeline. llustration of the main steps performed to generate the identification and annotation of repetitive elements genomes.

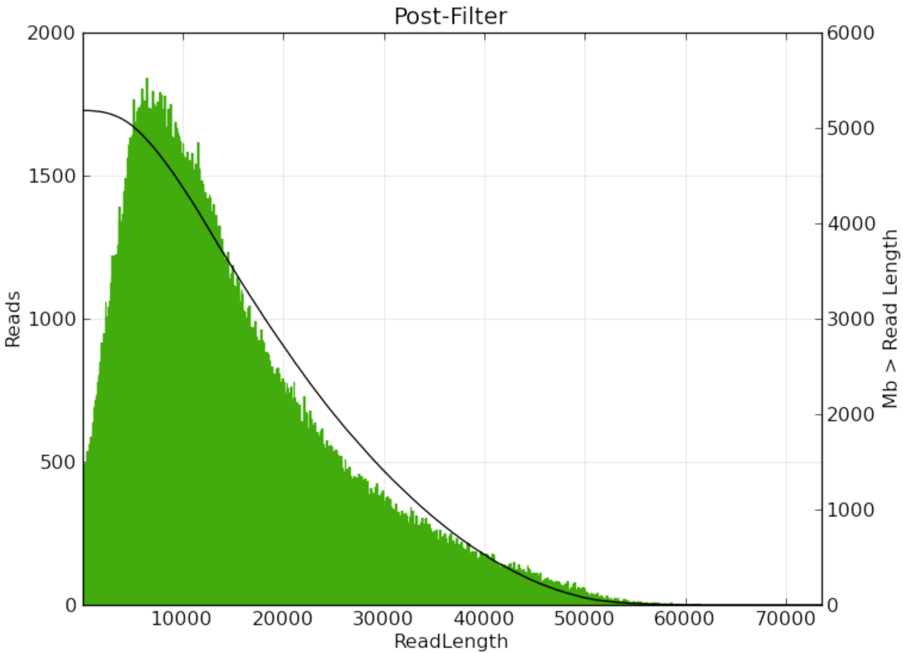

Fig. S3. Length distribution of filtered reads from TCC strain

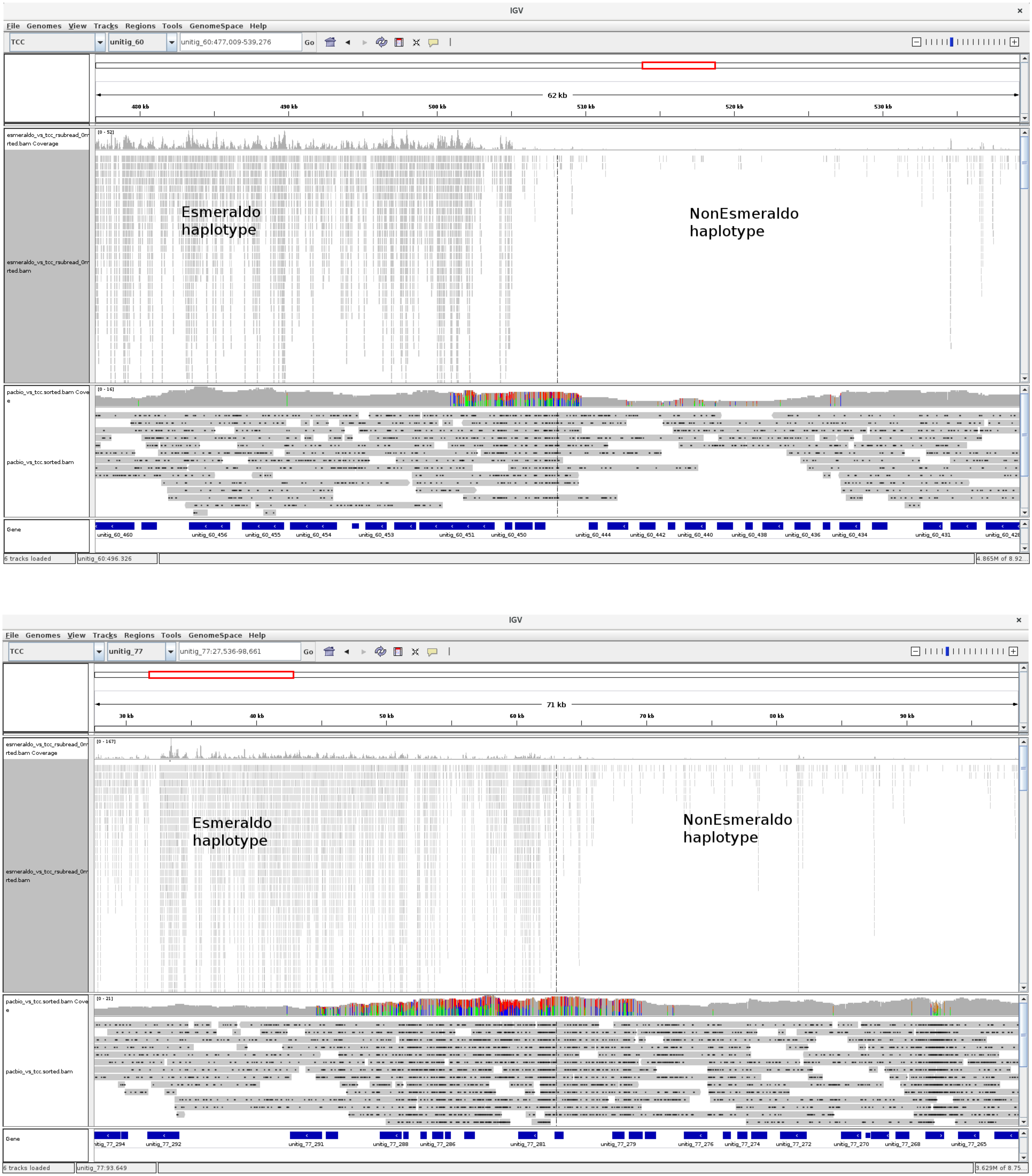

Fig. S4. Examples of "chimerical" contigs. It is exemplified the problems of mapping PacBio reads including high abundance of mismatches only at the artificial "recombination" point

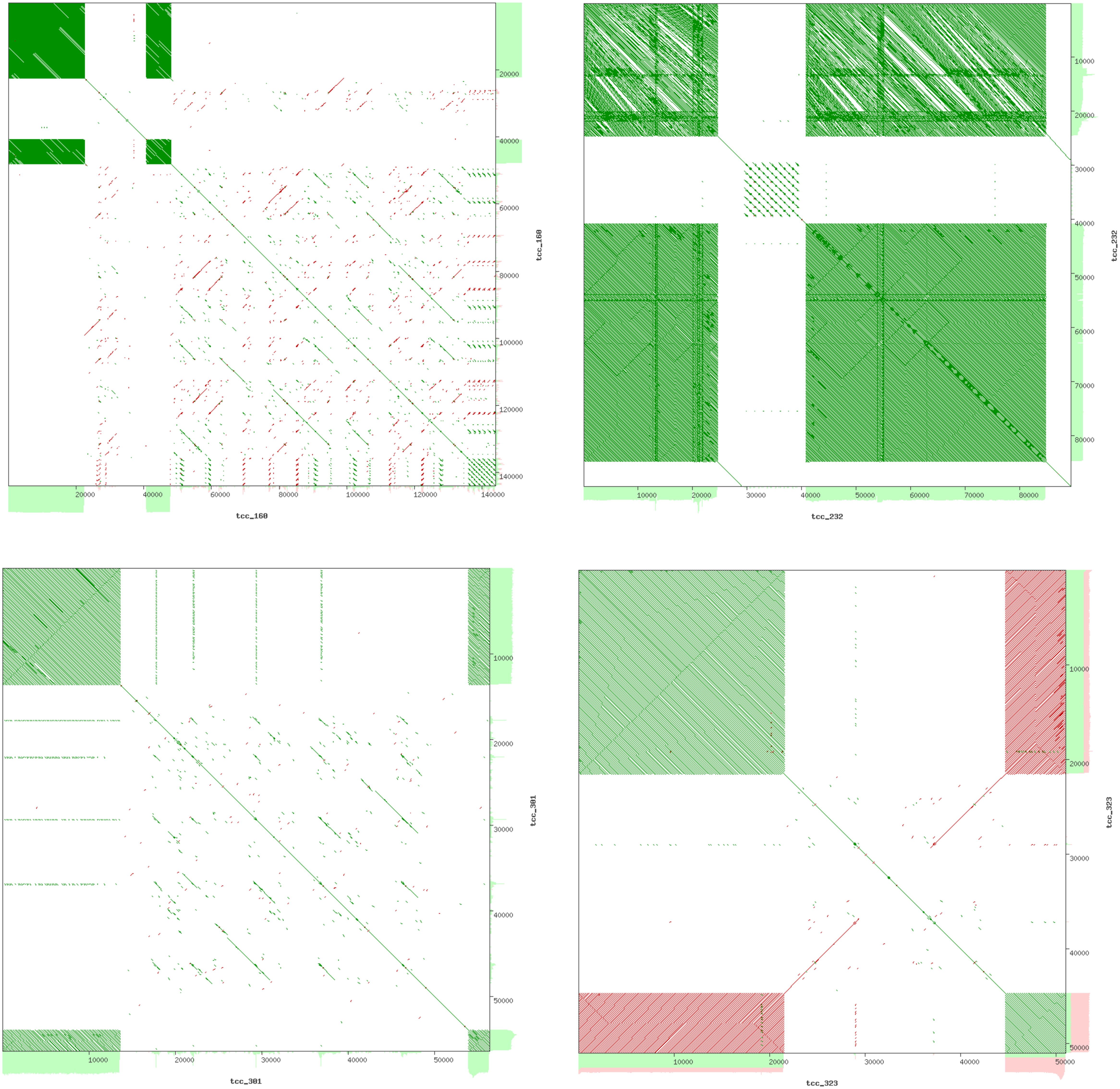

Fig. S5. Satellite visualization. Dotplots (YASS) of self-comparative mapping of contig including edge and internal regions of satellites in T. cruzi TCC.
